# 

**Published:** 1858-06

**Authors:** 


### INDEX TO VOLUME FOURTEENTH.

For original communications, look for the names of the authors as well as the title of the subjects.

For selected and editorial articles, look for the titles of the subjects only.

For notices of books, look for the names of the authors.

### ILLUSTRATIONS.

PAGE.

Inversion of the Uterus,......................................235, 236, 237

The Burges' Thigh Splint and Fracture Bed,..............................331

### A

PAGE.

Abortion between the Fourth and Fifth Months of Pregnancy; supposed to be due to the Absorbtion of Spirits of Turpentine. By Dr. Hawley, (Buff. Med. Assoc.)...... 469

Aborigines, a New Tribe, without Hair,.......................... 640

Acétone. A New Anæsthetic Agent, 381

Advertisement of the Journal,....... 45

Albumen in the Urine, Means of its Detection,..................... 38

Alley, Dr. William B., Retention of Urine,.......................... 139

American Med. Association, Eleventh Annual Meeting of,............. 46

American Medical Association,...... 704

" " " Meeting of, at Washington, May, 1858, Letter from a "Delegate from New York," ........................ 186

Amputation by a New Method, (M. Maisonneuve,) ................ 415

Anæsthesia and Anæsthetics,........ 497

Anæsthesia, General, Case of, (Dr. Klaatsch,)........................ 300

Anæsthesia by Compression,........ 382

PAGE.

Anæsthetics, (London Lancet,)..... 434

Aneurism of the Arteria Innominata, Treated by Pressure on the Distal Side, (Dr. Edwards,)............ 297

Anaphrodisiac Properties attributed to Belladonna,................. 480

Aorta, Case of Aneurism of. By Dr. Wm. Gould,...................... 106

Apoplexy, Case of, during Pregnancy. By Dr. White, (Buff. Med. Assoc.) ......................... 212

Asthma, Case of. By Dr. Flint, (Buff. Med. Assoc.) ..................... 341

Ash Tea in Rattlesnake Bite,....... 492

Auscultation in Boston in 1793, .... 124

Avery, Dr. La Fayette, Case of Removal of both Testes, for Spermatorrhœa, by a Botanic Doctor. Case of Death from Chloroform,........ 583

### B

Back Numbers of Journals, ......... 384

Barclay on Medical Diagnosis, ..... 166

Batchelder on Paralysis of Motion,. 225

Belladonna in Juvenile Incontenence of Urine,......................... 371

PAGE.

Bibron's Antidote to the Poison of the Rattlesnake, Experiments with, 33

Bibron's Antidote, Experiments with, by Dr. A. M. Sebal, ........ 299

Bigelow on Nature in Disease, ..... 698

Biliary Calculi, Specimens of. By Dr. Hamilton, (Buff. Med. Assoc.) 17

Blockley Hospital, ................ 198

Blood Corpuscles, Changes in the amount of, by the Administration of Cod Liver Oil, ................ 559

Boardman, Dr. Jno., Case of Poisoning by Corrosive Sublimate, ...... 196

Bowen, Dr. Charles A., Case of Dislocation of the Head of the Femur into the Obturator Foramen, Reduced by Reid's Method, ......... 585

Books and Pamphlets Received, .... 378

Brain, Disease of. By Dr. Gould, (Buff. Med. Assoc.) ............ 660

Breast, Specimen of Disease of. By Dr. Hamilton, (Buff. Med. Assoc.) 109

Braun on Uræmic Convulsions of Pregnancy, .................... 543

Bright's Disease, Cessation of the Elimination of Odors as a Sign of, 609

Brodie. Mind and Matter, ......... 345

Bucknell and Tuke on Insanity, .... 408

Business Department of our Journal, 437

Burning of the Quarantine Buildings, 316

Burges' Thigh Splint and Fracture Bed, ......................... 330

Buffalo Medical College, ........61, 316

" " " Opening of the Session, ...............374, 439

" " " Annual Commencement, .................... 638

" " " New Appointments in, .................. 640

Buffalo General Hospital, .......... 180

" " " Article on, in the Peninsular and Independent, . 373

" " " Report of, .. 573

Buffalo Hospital of the Sisters of Charity, Report of Cases in the Medical Wards. By A. W. Nichols, M. D., ...................... 39

Buffalo Med. Association, Abstract of Proceedings of, ............... 17

" " .............. 108

PAGE.

Buffalo Med. Association, Abstract of Proceedings of, ........ ........ 157

" " .............. 199

" " .............. 261

" " .............. 335

" " .............. 404

" " .............. 467

" " .............. 537

" " .............. 588

" " .............. 660

" " .............. 733

Buffalo Medical Association, Action of, in Regard to Criminal Abortions, ........................ 372

C

Calculus of Oxalate of Lime, Specimen of. By Dr. Wyckoff, (Buff. Med. Assoc.) .................. 473

Catalepsy, Case of, (Dr. Buchanan,). 171

Carnochan's Contribution to Operative Surgery, ............... ... 229

Cases from Dr. Hamilton's Clinic. Reported by Dr. Jno. Boardman, (Hydrocele—Hare-lip,) ......... 460

" " (Encysted Tarsal Tumors—Nasal Polypi,) ....... 530

" " (Nævi,) ......... 577

" " (Frac. of Femur,) 570

Carate, a Non-classified Disease of the Skin, ...................... 686

Carbonic Acid, Inhalation of, as an Anæsthetic, ................... 699

" " Quantity evolved from the Lungs under the Influence of various Agents, ................ 562

Cent of the Old Coinage, Case of Accidental Swallowing of. By O. C. Gibbs, M. D., .................. 27

Cerebro-Spinal Meningitis. By J. W. Craig, M. D., .............. 65

Chloroform, Deaths from. By La Fayette Avery, M. D., ........... 583

Chloroform in Discriminating between Muscular Contraction and Coxalgia, ...................... 485

Chorea Sancti Viti, Annual Address of the retiring President of the Buff. Med. Association. By C. C. Wyckoff, M. D., ................ 734

PAGE.
Claims of the Medical Art to the Consideration of the Public at the Present day. By Theoph. Mack, M.D., 449
Clinical Study of the Heart-Sounds. By Austin Flint, M. D., 321
Convention of Teachers at Louisville, 670
Coxe on True or Membranous Croup, 506
Compression of the Carotid in Bleeding from the Nose, 493
Congenital Fissure of the Sternum, 448
Conservative Medicine. The Annual Address of the retiring President of the Buff. Med. Association. By Austin Flint, M. D., 135
Corrosive Sublimate, Case of Poisoning by. Reported by John Boardman, M. D., 196
Cow's Milk, How to Render it a more Suitable Food for Children, 357
Craig, Dr. J. W., Cerebro-Spinal Meningitis, 65
Craneotomy without Instruments—Case of Difficult Labor—Monster. By N. L. North, M. D., 149
Criminal Abortions, 247, 309
" " Letter detailing a case of, 445
" " Bible Argument Against, 697
Croup, Pseudo-membranous. Case by Dr. Hawley, (Buff. Med. Assoc.) 733
Croup, Pyogenic, Case of. By Dr. Rochester, (Buff. Med. Assoc.) 338
Crooks, Dr. John C. K., Case of Inflammation of the Spinal Cord, 511
Cutaneous Anæsthesia in Croup, 485

D

Dalton's Treatise on Human Physiology, 595
Da Costa, Dr. J. Translation of a Case of Fissure of the Sternum, permitting Exam. of the Heart, 151
Death of the Lower Extremity from a Tight Bandage. Letter from Dr. R. H. Dalton to Dr. Hamilton, 193
Death of the Foot produced by a Tight Bandage, 32
Death of Prof. J. K. Mitchell, 63
Death of Dr. Robinson, of Detroit, 64

PAGE.
Death of Dr. H. H. Bissell, and of Prof. Hare, 318
Death of Dr. Billings, Prof. Mauthner, Johannes Müller, Dr. Wm. Gregory, Dr. Robert Harrison, Sir Jas. McGregor, Sir Phillip Crampton, Dr. John Snow, Dr. Merei, and Dr. Elisha Townsend, 375
Death of M. Bérard and Dr. Samuel Sawyer, Dr. Richard Bright, Dr. Canton, Dr. J. S. Duval and Dr. David Uhl, 575
Death of Drs. Geo. M. Newton, Peter C. Gaillard, Thos. D. Mütter, G. C. Goble, Prof. Ellet, Dr. Edw'd Hudson and Dr. George Abbott, 703
Death from Want of Sleep, 702
Deaths, Monthly Record of, (April,) 31
" " " (May,) 113
" " " (June,) 167
" " " (July,) 228
" " " (August,) 295
" " " (September,) 354
" " " (October,) 413
" " " (November,) 479
" " " (December,) 546
" " " (January,) 607
" " " (February,) 677
" " " (March,) 000
De La Cossitt, Dr. H., Case of Strangulated Inguinal Hernia successfully Treated, 586
Diabetes, Rationale of Saccharine Treatment of, 416
Diabetes, Etiology of, (Dr. Rees,) 115
Dislocation of the Head of the Femur into the Obturator Foramen in a Child, two years and three months old. Reduction by Dr. Reid's Method. By Chas. A. Bowen, M.D. 585
Dislocations of the Femur. Reduction by Manipulation. Fragments of the Literature of. By Frank H. Hamilton, M. D., 1
Dislocation of the Sternal Extremity of the Clavicle. By Dr. Rochester, (Buff. Med. Assoc.) 262
Digestion, Experiments on, (Harley,) 610
Dunglison, Dr. Richard J. Observations on the Deaf and Dumb, 575

### E

PAGE.
Editorial Courtesies, ... 384
Emmerson's Magazine and Putnam's Monthly, ... 127
Epicystotomy, (E. Noeggerath, M.D.) 35
Epilepsy, Cases compiled from Dr. Hamilton's Notes, ... 400
Epidemic Sore Throat in Albany, (Dr. Willard,) ... 495
Erie Co. Med. Society, Semi-Annual Meeting of, ... 123
" " " Annual Meeting of, 512
" " " " " 570
Erichsen's Science and Art of Surg., 676
Erysipelas, Cases of. By Dr. Gould, (Buff. Med. Assoc.,) ... 404
Erysipelas, Profound or Phlegmonoid. By George Niemeier, M. D., ... 98
Erysipelas, Its Constitutional Origin and Treatment. Annual Oration before the Erie Co. Med. Society. By C. C. F. Gay, M. D., ... 662
Eve, Dr. Jos. A., on Diseases of the Cervix Uteri, ... 254
Excision of the Inferior Maxillary Nerve for Neuralgia. By F. H. Hamilton, M. D., ... 148
Eye, Extirpation of, (Buff. Med. Association,) ... 18

### F

Falcony's Powder for Preserving Dead Bodies, ... 689
Fibula, Excision of, for Fibro-Cartilaginous Degeneration of the Bone, 548
Flint, Prof. Austin, Annual Address of the Retiring President of the Buff. Med. Association. On Conservative Medicine, ... 135
" " " Clinical Study of the Heart-Sounds, ... 321, 385
" " " Letter from New Orleans, ... 513
" " " Report of Cases of Pneumonia Treated at the Charity Hospital, New Orleans, ... 705
Flint, Dr. Austin, Jr., Case of Injury to the Fingers from a Hay Cutting Machine, ... 150
" " " Resolutions of Class of 1859, ... 639
PAGE.
Flint, Dr. Austin, Jr., Translation of Balbiani on Sexual Generation in Infusoria, ... 257
" " " The Determination by Chemical Reagents of the Identity or Non-Identity of Specimens of Writing Ink on Paper, .. 525
Forearm, Injury of. Case by Dr. Flint, Jr., (Buff. Med. Assoc.) ... 23
Forbes, Sir John. Nature and Art in the Cure of Disease, ... 411
Fracture of the Astragulus. By Bernard Monahan, M. D., ... 266
Fracture, Treatment of, by Plaster of Paris, at the Berlin and Vienna Hospitals, ... 429

### G

Gay, Dr. C. C. F., Erysipelas. Annual Oration before the Erie Co. Medical Society, ... 662
Gibbs, Dr. O. C., Strichnine in Sciatica, 333
" " Case of Swallowing of a Cent of the Old Coinage, ... 31
Glands, Cervical, Enlargement of. By Dr. Hamilton, (Buff. Med. As.) 23
Gleet, Treatment of, by Local Remedies. (Wm. Otterson, M. D.,) ... 423
" Treatment of, by Local Rem., 557
Glass, Extracted from the Arm, ... 569
Gonorrhœa, Case of. By Dr. Jansen, (Buff. Med. Assoc.,) ... 593
Gould, Dr. Wm., Case of Aneurism of the Arch of the Aorta, ... 106
Gorilla, ... 707
Green, Favorite Prescriptions of Living American Practitioners, ... 606
Green, Dr. Horace, ... 572
" " and N. Y. Academy, 619
Gun-shot Wound of Sup. Extremity, 694

### H

Hamilton, Prof. F. H., Reduction of Dislocations of Femur by Manip., 1
" Frac. of Shaft of Femur, .. 129
" Excision of Inf. Max. Nerve for Neuralgia, ... 148
" Burges' Thigh Splint, etc., 330
Hare-Lip, New Operation, (A. Duke,) 547

PAGE.
Hare-Lip, Suffocation following the Operation, (Dr. Jacobi,)........ 547
Hand, Injury to, from Hay-Cutting Mach. By Austin Flint, Jr., M.D., 150
Hæmorrhage from Ear as a Sign of Fracture at Base of Skull. By Dr. Ham, (Buff. Med. Assoc.)........ 199
Heart, Nerves of, ................ 39
Heart, Case of M. Groux. By J. Da Costa, M. D.,.................. 151
Heart Sounds, Clinical Study of the. By Prof. Austin Flint, M. D., .... 385
Hernia, Case of Strangulated Inguinal, successfully treated. By H. De La Cossitt, M. D.,............ 586
Hip, Dislocation of. By W. W. Jones, M. D.,...................... 103
Hip, Obscure Injury of. Letter from Dr. Wm. B. Sprague,............ 536
Hydro-pneumothorax. Paracentesis and Iodine Injections,........... 370
Hydrophobia from Bite of Pole Cat,. 553
" Case of Spontaneous,..... 684

I

Imperforate Rectum, Case of....... 441
Insanity and Crime, .............. 41
Influence of two Distinct Nerves in Blood of Glands, (Bernard,) ..... 481
Influence of Parents on Progeny,... 617
Inks, Chemical Examination of Specimens. By Austin Flint, Jr. M.D., 525
Iodine, New Modes of Administrat'n, 561
Iron Rod Pushed through Abdomen, 466

J

Jenner Monument, Inauguration of,. 123
Jones, Dr. W. W., Dislocation of Hip, 103

L

Lacerated Perineum, Operation for. By Dr. White, (Buff. Med. Assoc.) 405
Lallemand and Wilson on Spermatorrhœa, ...................... 407
Lemon, Dr., Compliment to,........ 693
Letter from Prof. Flint,........... 513
Literary Piracy, .................. 383
Louisville University, Changes,..... 64
Lumbrici in Biliary Ducts, (Bonfils,) 491

M PAGE.

Maine Med. & Surg. Reporter, ..... 127
Malignant Pustule, (Virchow,)..... 704
Marshall Hall Method in the case of Child apparently Still-born. By Dr. Lemon, (Buff. Med. Assoc.).. 111
Malformation, Case of. By W. A. Green, M. D.,................. 246
Malformation, Case of,............ 568
Massachusetts Med. Soc., Prizes of, . 381
Mack, Dr. Theophalus, Claims of the Medical Profession, etc.,......... 449
" Monthly "*Esquisse*" of Parisian Medicine and Surgery, 652, 725
Mathews, A. I., List of Drugs, etc.,.. 511
Mammoth Cave of Kentucky,....... 668
Medical Journal at Athens, Greece,.. 191
Medical Journal of South Carolina,. 256
Medical and Surgical Reporter,..... 320
Medical Student, ................. 502
Med. Society of South Western New York, Annual Meeting of, ....... 443
Mediastinal Tumor. By Dr. Ring, (Buff. Med. Assoc.) ............ 211
Meigs, Dr. J. Aitken. Hints to Craniographers,..................... 576
Mercer, Dr. Alfred. Partial Dislocation of Shoulder-Joint, etc.,...... 641
Mineral Waters of St. Catherines,... 183
Monahan, Dr. Bernard. Fracture of the Astragalus,................ 266
Monstrosity, Case of. By Dr. White, (Buff. Med. Assoc.)............. 537
Morland on the Urinary Organs,.... 540
Muscle, A Singular One, (Marion Howard, M. D.,)............... 432
Mütter Museum,.................. 700

N

Nausea, Excessive, during Pregnancy, Cauterization of Cervix, ......... 369
Nashville Medical Recorder,....... 383
New Volume,.................... 44
N. Y. State Inebriate Asylum, ..... 440
New York State Medical Society, Annual Meeting of,............. 627
New York Medical Press, ......... 510
New Arrangements, .............. 639
New Medical Exchanges,.......... 571
New Orleans School of Medicine, Appointment of Prof. Flint,...... 191

PAGE.

Needle carried in the Foot for three Months. By Dr. Boardman, (Buff. Med. Assoc.) .......... 473
Needle passing in at the Little Toe and out at the Great Toe. By Dr. Treat, (Buff. Med. Assoc.) ....... 473
Neimeier, Dr. George. Profound or Phlegmonoid Erysipelas, ........ 98
Nice Thing for Invalids, ........... 59
Nitric Acid in Treatment of Hæmorrhoids. By Dr. Gould, (Buff. Med. Assoc.) .......... 335
Nichols, Dr. A. W., Cases in Medical Wards of the Buffalo Hospital of the Sisters of Charity, ........... 99
North American Medical Reporter, . 447
North, Dr. N. L., Difficult Labor—Monster—Craneot. without Inst., . 149

O

Obstetrical Case, Remarkable, ..... 691
Obstetrics, Case of, with Lax Condition of the Abdominal Walls. By Dr. White, (Buff. Med. Assoc.) ... 209
Obstetrics, Case of Deformity of the Pelvis. By Dr. White, (Buff. Med. Assoc.) .......... 265
Obstruction of the Bowels Relieved by Copious Injections, (Dr. Gibbs,) 433
"Od," A New Imponderable Agent, 379
Ontario Medical Society, Annual Meeting of, .......... 377
Ossification of the Cricoid and Thyroid Cartilages, Case of, by Dr. Lemon, (Buff. Med. Assoc.) ..... 405
Oxygen in Venus Blood, (Bernard,) 611
Ozonometer, .......... 560

P

Parturition without Pain, Case of, by Dr. Miner, (Buff. Med. Assoc.) .... 212
Pacific Med. and Surg. Journal, ..60, 640
Parisian Med. and Surgery, Monthly "*Esquisse*" of. By Theophalus Mack, M. D., .......... 652, 725
Palmer's Artificial Leg, .......... 126
Palmer's Lectures on the Sulphate of Quinia, .......... 255
Palpitation of the Heart, (Kölliker,) 174
Paine's Institutes of Medicine, ..... 213

PAGE.

Paine's Vitality and Remed. Agents, 255
Painless Caustic, .......... 357
Penetrating Wound of the Abdomen, Case of. By Dr. Rankin, (Buff. Med. Assoc.) .......... 467
Perforation of the Pericardium by Echinococci, .......... 470
Perineuron, A New Element in Nerves, (Robin,) .......... 41
Perineum, Case of Laceration of. By Dr. White, (Buff. Med. Assoc.) ... 159
Peritonitis, Traumatic, Case of. By Dr. Rochester, (Buff. Med. Assoc.) 108
Phlebolites, Specimens of. By Dr. Hamilton, (Buff. Med. Assoc,) .... 22
Physician's Charitable Fund Assoc., 189
Physician's Diary for 1859, ........ 448
Plates Illustrative of Wilson on Diseases of the Skin, .......... 128
Plague at Bengazi, .......... 382
Placenta, Removal in Early Months of Pregnancy, (O. C. Gibbs, M. D.,) 679
Pneumonia treated with Quinia, .... 365
" Cases of, Treated at the Charity Hospital, New Orleans. By Austin Flint, M. D., .......... 705
Polypus of the Œsophagus, (Prof. Middelderpff,) .......... 552
Polypoid Excrescences in the Uterus. By Dr. White, (Buff. Med. Assoc.) 405
Presentation of Medical Accounts. Report of the Committee of the Buffalo Medical Association, ..... 157
Progress of Invention, .......... 42
Professorial Changes, ..........319, 511
Private Medical Instruction, ....... 638
Properties and Uses of the Red and Black Blood, (Brown Séquard,) ... 360
Puerperal Convulsions, Case of. By Dr. White, (Buff. Med. Assoc.) .... 165
Purpura Hæmorrhagica. By Dr. Butler, (Buff. Med. Assoc.) .......... 592

Q

Quinine Makers, Diseases of, ....... 368
Quackery at Home and Abroad, .... 563

R

Ranula, New Operation for, ........ 432
Rape, Microscope in a case of, (A. F. Sawyer, M. D.,) .......... 169

PAGE.
Reed, Inaugural, Dissertation on Strychnia, .................... 353
Resignations and Appointments,... 251
Retention of Urine. By Wm. B. Alley, M. D.,.................... 139
Respiratory Movements of an Infant in Utero, (Dr. Schultze,).......... 701
Ricord and Hunter on Venereal, ... 699
Reid on Ventilation in American Dwellings,..................... 293
Robin, Dr. Chas., Appointment in the Academy of Medicine,........ 174
Rochester, Dr. Thos. F., Tracheotomy in Pseudo-Membranous Croup, 81
Rupture of the Uterus, Case of. By Dr. Butler, (Buff. Med. Assoc.,... 342

S

Santonine, (M. Martini,)........... 551
Sarsaparilla, Therapeutic Prop. of,.. 561
Savannah Journal of Medicine,..... 62
Saccharine Urine, Various Tests for, (Becquerel,) .................. 241
Salivation by a Homœopathist. By Dr. Gould, (Buff. Med. Assoc.) ... 473
Sciatica, Strichnine in. By O. C. Gibbs, M. D.,................. 333
Scrotum, Bony Tumor of, (John G. Kerr, M. D.,) .................. 39
Sexual Generation in Infusoria, by Dr. E. G. Balbiani. Translated by Austin Flint, Jr., M. D., .......... 257
Shoulder-Joint, Partial Dislocation, etc. By Alfred Mercer, M. D.,... 641
Shelby Medical College,........... 319
Sims on Silver Sutures,............ 221
Smith, Tyler, on Obstetrics,........ 675
Smith, Dr. E. P., Case of Traumatic Stricture, ...................... 395
Spina Ventosa, Case of. By Dr. Rochester, (Buff. Med. Assoc.,) ..... 405
Spinal Cord, Case of Inflammation of. By John C. K. Crooks, M. D., .... 521
Sprague, Dr. W. B., Case of Obscure Injury of the Hip, ............. 536
Spermatorrhœa, Removal of both Testes for, by a Botanic. Reported by La Fayette Avery, M. D.,..... 583
Starch from the Animal Kingdom, (Dr. Avery,) ................... 421

PAGE.
Stricture, Traumatic, of Ten Years' Standing, complicated with Fistula Perinei. By E. P. Smith, M. D.,. 395
Sugar in the Urine, Error in the Determination of, ................ 495
Sugar in the Urine, Means of Detecting, .......................... 38
Sulphuric Ether. Suit of Dr. Morton, 313
Suture of the Extensor Tendons of the Fingers,.................... 356
Surgical Operations, After Treat. of, 371
Supra Renal Capsules, Pathology of, 175
Swill Milk,....................... 119
Syphilization, ................... 43
Symetrical Tumors over the Olecranon. By A. M. Leonard, M. D.,.. 62
Symetrical Tumors. Letter from Dr. W. Young, M. D.,........... 190
Syrup of the Hypophosphites,....... 315

T

Tabes Mesenterica, Post-mortem Appearances in a Case of, ... ...... 694
Tape Worm. By Dr. Wyckoff, (Buff. Med. Assoc.)............. 661
Testicle, Displacement of, into the Perineum—Plastic Operation Unsuccessful—Castration, .......... 555
Tetanus, Case of. By Dr. White, (Buff. Med, Assoc.)............. 207
Tetanus, Report of Dr. Miner, (Buff. Med. Assoc.).................. 588
Tetanus, Traumatic, Case. By Dr. Miner, (Buff. Med. Assoc.)........ 207
Thayer's Fluid Extracts,........... 319
Thomas on Prolapsus of the Funis,.. 255
Tilden's Pharmacutic Sugar Coated Pills and Granules, ............. 320
To Our Subscribers,................ 320
Trachea, Case of Inflammation of, Fibrous Cast Thrown off,........ 373
Tracheotomy, Statistics of,.......... 428
Tracheotomy in Pseudo-Membranous Croup. By Dr. Thos. F. Rochester, (Buff. Med. Assoc.) ...... 81
Trachea, Rupture of, by a Fall, (Dr. Atlee,) ....................... 43
Transactions of the New York State Med. Society for 1858, .......... 275
Truss, Dr. Reynold's New Belt,.... 315

PAGE.
Trial for Rape in Montreal, ........ 494
*Ts'ün T'ai San Lun.* A New Description of the Entire Body. By Benj. Hobson, M.D., Canton, China, 40
Tuberculosis, Proximate Cause and Specific Remedy of, (Churchill,).. 243
Tubing of the Glottis, (Bouchut,)... 485
Typhus, Exanthematic, (Translated from Wunderlich,)............. 396

U

Ulna, Fracture of the Shaft of. By Prof. F. H. Hamilton, ............ 129
Umbilical Hernia in a Newly Delivered Female. By Dr. Rochester, (Buff. Med. Assoc.)............. 538
Umbilical Hernia, Case of Rupture of —Peritonitis—Opium—Recovery, 425
Umbilical Hæmorrhage. By Dr. Wyckoff, (Buff. Med. Assoc.).... 112
Uterus, Cancer of. By Dr. Hamilton, (Buff. Med. Assoc.)............. 18
Uterine Hæmorrhage. By Dr. Rochester, (Buff. Med. Assoc.)....... 25
Uterus, Case of Inversion, Reduced after Six Months. By James P. White, M. D.,.............25, 230, 251
Uterus, Complete Inversion of, Nearly Twelve Years' Standing, Reduced by Dr. Dr. Tyler Smith,... 241

PAGE.
Uterus, Inversion of, Fifteen Years' Standing, Reduced by Dr. Jas. P. White, (Buff. Med. Assoc.) ..... 264
Uterus of a Female who died while Menstruating, ................. 422

V

Varicocele,........................ 686
Vaginal Stethescope,.............. 61
Vaccination. By Dr. T. F. Rochester,, (Buff. Med. Assoc.).......... 539
Variola, Discussion of, in the Buff. Med. Assoc..................... 471
Venous Blood, Variations in Color of, (Bernard,) .................... 358

W

Warts of the Vulva. By Dr. Hamilton, (Buff. Med. Assoc.) .......... 163
Watson's Lectures on Practice,...... 352
Warning to Invalids Visiting the Island of Cuba,.................... 435
West on Diseases of Women,........ 473
White Swellings, (Bonnet,)........ 485
Wilson's Human Anatomy, ......... 352
Wines, Medical Uses of,............. 301
Wyckoff, Dr. C. C., Chorea Sancti Viti. The Annual Address of the Retiring President of the Buffalo Medical Association, ............ 734

We have received communications from Drs. Hamilton, Rochester, Lemon Nichols, Jones, Crooks and Niemeier, which will have an early insertion. Also a review of Payne's Institutes of Medicine, which is also excluded.

*Vaginal Stethescope.*—In a letter from Edinburgh, contained in a late number of the Boston Journal, an account of a vaginal stethescope invented by Prof. Keiller, to be applied to the stethescopic diagnosis of pregnancy; we extract a portion of the letter referring to Prof. Keiller and his invention:

Under the clinical instruction of Prof. Keiller is *the* place to gather practical, reliable, and useful instruction in the department of female diseases. He is perfectly free from ostentation, is gentlemanly and kind to his students and the patients; and while he does not neglect or abjure what is known to be good, he is eagerly searching for new means of diagnosis and for improvement in the modes of treatment. He has invented what he calls a vaginal stethescope, with which can be diagnosticated intra-uterine life at the second or third month, long before it can be discovered by the abdominal examination and the ordinary stethoscope. The instrument is, in material and shape, externally like the stethoscope used for the diagnosis of thoracic diseases, &c., but is solid, rather longer, and larger. It is passed up the vagina and its end pressed against the os uteri. With the instrument in this position, a sound is distinctly heard in the early months of pregnancy—more indistinct in later months—like the ordinary placenta souffle, or like that sometimes heard in an intra-pelvic fibrous tumor. The age, history and health of the patient, the condition of the menses, breasts, &c., must aid in the diagnosis as to the *nature* of this intra-uterine tumor. This stethoscope aids as to the fact of its *presence.*

---

*Buffalo Medical College.*—The Demonstratorship of Anatomy in the Buffalo Medical College, made vacant by the appointment of Dr. Nichols as lecturer on that branch, has been filled by the appointment of Dr. Benjamin H. Lemon.

Dr. Lemon is a thorough anatomist, and, we have no doubt, will fill his new position with entire satisfaction to the Faculty and class, and with credit to himself.

LOCKPORT, May 31st, 1856.

PROF. HAMILTON:

Dear Sir,—I send you the followfng report of a case that occurred in my practice, and which may possess interest enough to entitle it to a more extended publicity. You are at liberty to do with it as you see fit:

In the summer of 1853, Mr. Monroe Levally, wagon-maker, aged 46, called at my office and requested me to examine his arm. I did so, and found immediately over the point of the olecoron process an encysted tumor of the size of a robin's egg, and which made its appearance a few days previous. I made a few incisions into the sack with an abcess lancet, and obtained about ʒiii of a thick fluid. Introduced into the sack a pledgit of lint, and in a few days a perfect cure was effected.

But the next week I was not a little surprised, when he returned, with *another tumor* on the *other* arm, an exact counterpart of the prevlous one in every particular. It made its appearance in the same manner; in locality it was the same, (except on the other arm,) of the same size and form. I treated in the same manner, and the result was the same.

How far this goes to prove the existence of a sympathitic action between the corresponding points of the human system, we leave for others to decide; but it does certainly go far to prove that, under certain circumstances, one elbow at least has some sympathy with the other.

Yours, ever,

A. M. LEONARD.

DR. FLINT,—"Symmetrical diseases" are known to occur often in eruptive affections, and occasionally in rheumatic and syphylitic affections, but this is the only example which I have known where an encysted tumor, or a true tumor of any kind, has illustrated this curious law of affinities between opposite portions of the body.

Yours,

F. H. HAMILTON.

---

*The Savannah Journal of Medicine.*—We have received the first number of this Journal, issued in May. The editors are J. S. Sullivan, M. D., Josiah Harris, M. D., Prof. of Physiology in the Savannah Medical College, and R. D. Arnold, M. D., Prof. of Theory and Practice of Medicine in the Savannah Medical College, Associate Editor. This Journal will be issued bi-monthly, and contains seventy-two pages. The first number presents a fine appearance, and contains many valuable articles. We welcome it to our exchange list.

We have also received the first number of the *Oglethorpe Medical and Surgical Journal*, issued bi-monthly, and edited by Drs. Byrd and Steele, of which we can also speak in no less complimentary terms.

---

*Death of Prof. J. K. Mitchell.*—We announce with deep regret, the death of Prof. J. K. Mitchell, who has long held the chair of Practice in the Jefferson Medical College, and who is known to the profession as one of the most eminent teachers of that branch we have ever had among us. Prof. Mitchell has suffered of late years from partial hemiplegia, but has been able to fulfill the duties of his chair up to the close of the last session, when his health was better than it had been for some years. He was attacked, however, this spring, with pneumonia, which terminated fatally, taking from us one of our profession's noblest ornaments. Dr. Mitchell was distinguished for his devotion to polite literature as well as the cause of medical science; he was an honorable, high-minded man, and a kind and courtly gentleman of the old school. The vacancy has been filled by the appointment of Dr. Dickson, the eminent Professor of Practice at Charleston, who was unanimously recommended by the faculty.

We learn also from the North American Medico-Chirurgical Review, of the death of Benjamin Travers, the distinguished English surgeon; Dr. Forbes Royle, the author of a well known work on Materia Medica; Dr. I. K. Matther, of the University of Leipzig; Dr. Schoepf, of the University of Pesth; and M. L. Bandon, of Paris. We have also notice in the daily papers an announcement of the death of M. Chomel.

---

*Books and Pamphlets Received.*—We have received from the publisher, Barclay on Medical Diagnosis, Graham's Elements of Inorganic Chemistry, Wilson's Plates of Diseases of the Skin, the Transactions of the New York State Medical Society, Illinois State Medical Society; and from the author, Paine's Institutes of Medicine.

Reviews of these works have been crowded out by the Transactions of the

American Medical Association, but will appear as soon as possible. We have also received from the authors, the following pamphlets:

The Sulphate of Quinia, by A. B. Palmer, A. M., M. D.

Medical Opinion in the Parish Will Case, by Pliny Earle, M. D.

Silver Sutures in Surgery, by J. Marion Sims, M. D.

Diseases of the Cervix Uteri, by Joseph A, Eve, M. D.

Dysentery, its Pathology and Treatment, by Robt. Campbell, A. M., M. D. which will also receive early notice.

---

*Death of Dr. Robinson.*—We learn, as we go to press, of the death of Dr. L. G. Robinson, of Detroit, who was one of the editors of the Medical Independent, and afterwards of the consolidated Journal. Dr. Robinson was well known in the editorial world as an able and polished writer, and his death will be as much lamented by his editorial brethren, as by his large circle of friends at Detroit.

---

*Proposed Changes.—University of Louisville.*—We learn that Prof. J. B. Flint, who succeeded Prof. Gross in the chair of surgery, in the University of Louisville, has resigned, and Prof. Palmer, the accomplished teacher of Anatomy, in that institution, has been transferred the vacant chair.

We also learn that Prof. Miller, the venerable Professor of Obstetrics, has resigned. As yet we have heard no one mentioned as likely to fill either of the vacancies.

It is also rumored that the new medical school at Nashville, will soon go into operation, and that Dr. May, of Washington, is to be the Professor of Surgery.

---

*Nos.* 1 *and* 2 *of Vol. XI.*—We are entirely out of these numbers, with the exception of two or three which complete our full sets, and will cheerfully credit 50 cents to any subscriber who will send them to us.

